# How I approach membrane lung dysfunction in patients receiving ECMO

**DOI:** 10.1186/s13054-020-03388-2

**Published:** 2020-11-30

**Authors:** Bishoy Zakhary, Leen Vercaemst, Phillip Mason, Marta V. Antonini, Roberto Lorusso, Daniel Brodie

**Affiliations:** 1 Division of Pulmonary and Critical Care Medicine, Oregon Health and Science University, Portland, OR USA; 2 Department of Perfusion, University Hospital Gasthuisberg, Leuven, Belgium; 3 Department of Surgery, Brooke Army Medical Center, San Antonio, TX USA; 4 General ICU, I° Department of Anesthesia and Intensive Care, University Hospital of Parma, Parma, Italy; 5 Department of Biomedical, Metabolic, and Neural Sciences, University of Modena and Reggio Emilia, Modena, Italy; 6 Cardio-Thoracic Surgery Department, Heart and Vascular Centre, Maastricht University Medical Centre (MUMC), Cardiovascular Research Institute Maastricht (CARIM), Maastricht, The Netherlands; 7 Columbia University College of Physicians and Surgeons, New York-Presbyterian Hospital, New York, USA; 8 Center for Acute Respiratory Failure, New York-Presbyterian Hospital, New York, NY USA

## Introduction

With improvements in circuit technology and expanding supportive evidence, extracorporeal membrane oxygenation (ECMO) use has grown rapidly over the past decade [1]. Advances in pump and membrane lung (ML) design have led to simpler and more efficient circuits. Circuit-related complications, however, remain frequent and associated with considerable morbidity [2].

## Mechanisms of membrane lung dysfunction

The ML is responsible for oxygen uptake and carbon dioxide removal. The non-biologic surface of the ML activates inflammatory and coagulation pathways with thrombus formation, fibrinolysis, and leukocyte activation [3–5] leading to ML dysfunction. Activation of coagulation and fibrinolysis can precipitate systemic coagulopathy or hemolysis, while clot deposition can obstruct blood flow [6, 7]. Additionally, moisture buildup in the gas phase and protein and cellular debris accumulation in the blood phase may contribute to shunt and dead-space physiology, respectively, impairing gas exchange [8, 9]. These three categories—hematologic abnormalities, mechanical obstruction, and inadequate gas exchange—prompt the majority of ML exchanges.

## Membrane lung monitoring

### Hematologic profile

Monitoring of hematologic variables, including coagulation and hemolysis labs, can help identify the development of an ECMO coagulopathy or hemolysis.

### Pressure monitoring

The pressure drop across the ML (ΔP) is measured as (Additional file 1: Supplemental Figure):

$$\Delta P = P_{{{\text{Pre}}}} - P_{{{\text{Post}}}}$$

where *P*_Pre_ = pre-ML pressure, *P*_Post_ = post-ML pressure.

As clot forms in the ML, increases in resistance (*R*_ML_) are reflected as increases in Δ*P*. To correct for changes in blood flow rate (BFR), monitoring of ΔP normalized for BF rate (Δ*P*/BFR) more directly reflects *R*_ML_.

### Membrane lung gas transfer

Applying the Fick principle across the ML, oxygen (O_2_) transfer may be calculated as:

$$V^{\prime}{\text{O}}_{2} = {\text{BFR}}\left( {C_{{{\text{Post}}}} {\text{O}}_{2} {-}C_{{{\text{Pre}}}} {\text{O}}_{2} } \right)$$

where *V*′O_2_ = O_2_ transfer across the ML (mL/min), BFR = blood flow rate (L/min), *C*_*x*_O_2_ = O_2_ content of (pre-/post-ML) blood (mL/L) for

$$C_{x} {\text{O}}_{{2}} = {13}.{4} \cdot {\text{Hb}} \cdot S_{x} {\text{O}}_{{2}} + 0.0{3} \cdot P_{x} {\text{O}}_{{2}}$$

where Hb = hemoglobin (g/dL), *S*_*x*_O_2_ = O_2_ saturation of (pre-/post-ML) blood, *P*_*x*_O_2_ = O_2_ partial pressure of (pre-/post-ML) blood (mmHg).

Measurement of *V*′O_2_ provides an objective measure of oxygen transfer and can confirm ML dysfunction, when clinically indicated.

## Membrane lung dysfunction

Prompt recognition of ML dysfunction is vital for safety, allowing for elective replacement in a controlled manner. On the other hand, replacement of an adequately functioning device—requiring temporary cessation of ECMO support—places the patient at unnecessary risk while consuming a limited and expensive resource.

**Fig. 1:**
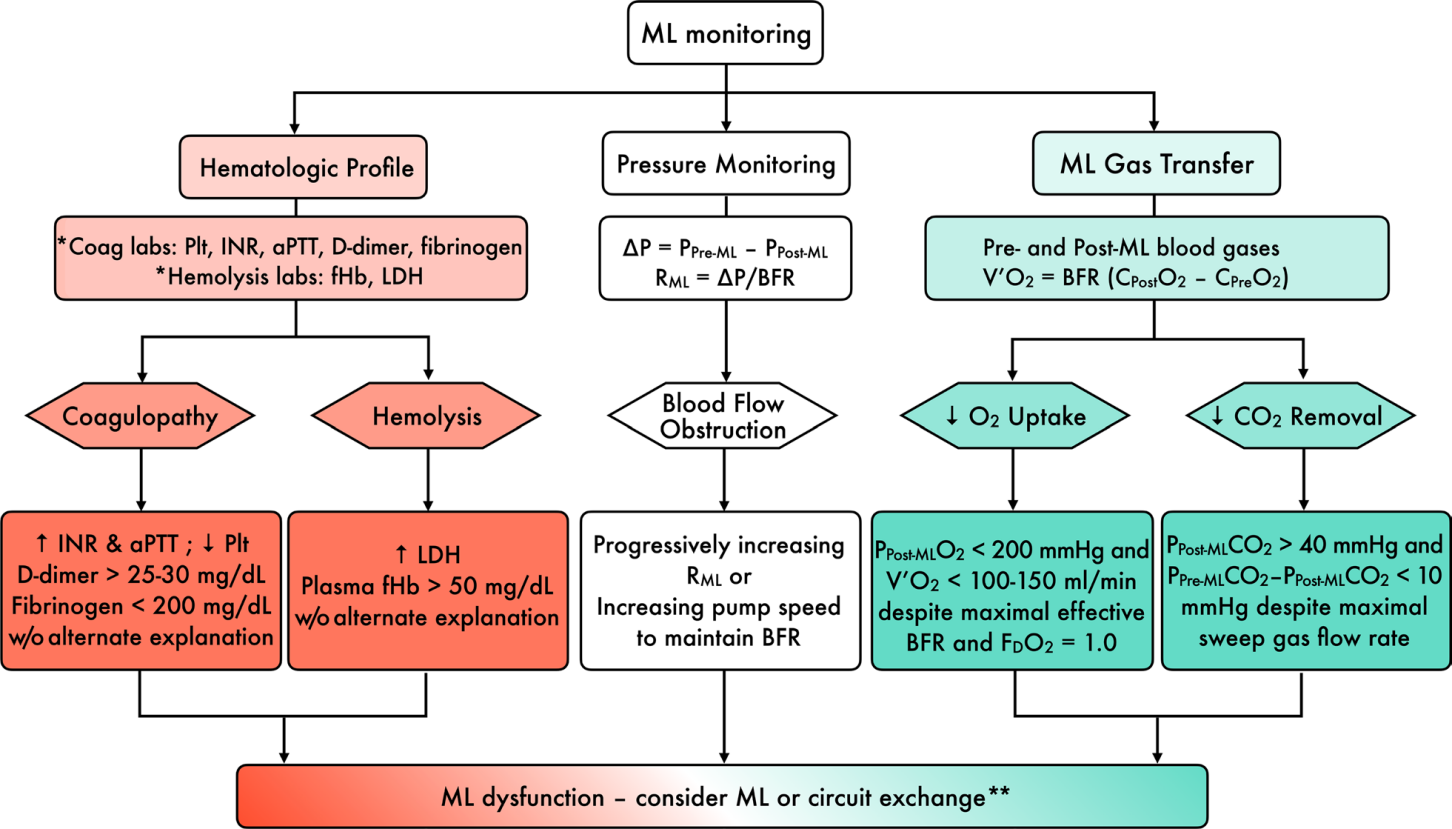
Algorithmic approach to membrane lung monitoring for membrane lung dysfunction. Cut-off values for consideration of ML exchange are suggested values based on author experience and should be considered in the context of the patient and dependence on ECMO support. Please refer to text for details. ML, membrane lung; Plt, platelet count; INR, international normalized ratio; aPTT, activated partial thromboplastin time; fHb, free hemoglobin; LDH, lactate dehydrogenase; Δ*P*, Pressure drop across the ML; P_Pre_, pre-ML pressure; P_Post_, post-ML pressure; *R*_ML_, resistance within the ML; BFR, blood flow rate; *V*′O_2_, membrane lung oxygen uptake; *C*_Pre_O_2_, O_2_ content of pre-ML blood; *C*_Post_O_2_, O_2_ content of post-ML blood; *P*_Pre_O_2_, partial pressure of pre-ML O_2_; *P*_Post_O_2_, partial pressure of post-ML O_2_; *P*_Pre_CO_2_, partial pressure of pre-ML CO_2_; *P*_Post_CO_2_, partial pressure of post-ML CO_2_. Flowchart is designed with adult ECMO patients in mind and may not be applicable to pediatric or neonatal patients. *Extent and frequency of coagulation and hemolysis lab monitoring is not well-established and will vary by center. Not all labs are required to diagnose coagulopathy or hemolysis. **When a ML fails, we recommend considering switching the entire circuit, rather than just the ML, if: (a) the ML and pump head are fused; (b) the ML dysfunction occurs in the setting of circuit-related coagulopathy; or (c) the ML dysfunction occurs in the setting of an older circuit (i.e., longer than 2 weeks). While the first is due to technical limitation, the latter aim to reduce the risk of ongoing or new circuit-related coagulopathy in circuits at risk for this phenomenon

Based on the pathophysiology of the ML, replacement may be required for one of three reasons: if there is (A) an associated hematologic abnormality, (B) an increasing obstruction to blood flow, or (C) inadequate gas exchange (Fig. 1).

### Hematologic abnormalities

The presence of an ECMO coagulopathy, typified by elevated clotting times, hypofibrinogenemia, thrombocytopenia, and elevated D-dimer without alternate explanation raises concern for *circuit-related coagulopathy* (CRC). Alternatively, evidence of hemolysis with elevated plasma-free hemoglobin, without alternate explanation, is concerning for circuit-related hemolysis. In both cases, the diagnosis is presumptive and only confirmed when values normalize after circuit exchange [6].

### Obstruction to blood flow

Increasing Δ*P*/BFR suggests increasing ML clot burden. As different MLs have different *R*_ML_, no cut-off values of Δ*P* define ML dysfunction and the trend should be carefully considered. A rapidly increasing Δ*P*, even if not associated with reduced gas exchange efficiency, is often a harbinger of impending ML failure and should prompt consideration of ML exchange. When ML pressures are not measured, an increasing pump speed requirement to maintain a stable BFR can serve as a surrogate for increasing ΔP, with the caveat that pump preload and afterload also affect this relationship.

### Inadequate oxygen uptake

Worsening oxygenation during ECMO should prompt quantification of oxygen transfer. When the ML is no longer able to meet patient oxygen demand, ML exchange is indicated. There are three important considerations in making this decision.

First, it is necessary that measured *V*′O_2_ is truly a maximal value. If circuit BFR is low, for example, the blood will be fully saturated early in the ML path and reserve will exist for additional oxygen transfer as BFR is increased. Similarly, if *C*_Pre_O_2_ is artificially elevated, due to high recirculation fraction or impaired tissue extraction, or if the fraction of delivered oxygen in the sweep gas (F_D_O_2_) is below 100%, the gradient driving oxygen transfer is reduced, and measured *V*′O_2_ may not represent maximal capacity. As such, BFR should be sufficiently high that further increases do not increase arterial saturation, recirculation fraction should be minimized, and ML F_D_O_2_ set to 100% to ensure an accurate assessment of maximal *V*′O_2_.

Second, though *P*_Post-ML_O_2_ less than 200 mmHg can suggest a failing ML [6], it is vital to calculate *V*′O_2_ for confirmation. In the setting of low *C*_Pre_O_2_ or high circuit BFR, blood exiting the ML may not be fully saturated, with low *P*_Post_O_2_, despite normal *V*′O_2_. In this case, if the ML is exchanged, the patient is placed at risk without subsequent improvement in oxygen delivery.

Finally, no absolute values diagnose inadequate oxygen transfer and clinical context is important. In general, however, in a patient with hypoxemia and a ML with maximal *V*′O_2_ < 100–150 mL/min, ML exchange is typically indicated.

### Inadequate carbon dioxide clearance

ML dysfunction can also manifest as inadequate CO_2_ clearance. Calculation of *V*′CO_2_ is not typically performed as it varies in a nonlinear fashion with sweep gas flow rate and requires sampling ML exhaust CO_2_ [10]. However, persistent *P*_Post-ML_CO_2_ greater than 40 mmHg [6] and clearance of less than 10 mmHg PCO_2_ between pre- and post-ML blood gases despite sweep gas flow rates of 10 L/min or greater is suggestive of ML dysfunction and ML exchange should be considered.

## Sudden membrane lung failure

While serial monitoring of the ML may identify markers of dysfunction and allow for elective exchange, acute ML failure is a potentially life-threatening event with unique considerations. Mechanisms to ensure optimal management are provided in the Additional file 2.

## Conclusion

The decision to exchange a ML is complex and without clear guidelines. In this manuscript, we outline a physiologic approach to troubleshooting this common yet high risk event.

## Supplementary information

**Additional file 1.** Membrane lung monitoring of pressure drop and oxygen transfer.

**Additional file 2.** Sudden Membrane Lung Failure.

## Data Availability

Not applicable.

## Abbreviations

ECMO: Extracorporeal membrane oxygenation
ML: Membrane lung
COVID-19: Coronavirus disease 2019
Δ*P*: Pressure drop across the ML
*P*_Pre_: Pre-ML pressure
*P*_Post_: Post-ML pressure
*R*_ML_: Resistance within the ML
BFR: Blood flow rate
*V*′O_2_: Membrane lung oxygen uptake
*C*_Pre_O_2_: O_2_ content of pre-ML blood
*C*_Post_O_2_: O_2_ content of post-ML blood
Hb: Hemoglobin
*S*_Pre_O_2_: Fractional O_2_ saturation of pre-ML blood
*S*_Post_O_2_: Fractional O_2_ saturation of post-ML blood
*P*_Pre_O_2_: Partial pressure of pre-ML O_2_
*P*_Post_O_2_: Partial pressure of post-ML O_2_
*V*′CO_2_: CO_2_ clearance across the ML
ExCO_2_: ML exhaust CO_2_
CRC: Circuit-related coagulopathy
F_D_O_2_: Membrane lung inlet oxygen fraction
*P*_Pre_CO_2_: Partial pressure of pre-ML CO_2_
*P*_Post_CO_2_: Partial pressure of post-ML CO_2_
Plt: Platelet count
INR: International normalized ratio
aPTT: Activated partial thromboplastin time
fHb: Free hemoglobin
LDH: Lactate dehydrogenase
