# Supplementary material for: How I approach membrane lung dysfunction in patients receiving ECMO
Source: Crit Care. 2020 Nov 30;24:671. doi: 10.1186/s13054-020-03388-2 (PMC7704102; doi:10.1186/s13054-020-03388-2)
Supplement: Supplementary file 1 — Additional file 1. Membrane lung monitoring of pressure drop and oxygen transfer. [file 13054_2020_3388_MOESM1_ESM.docx]

**Supplemental Figure**


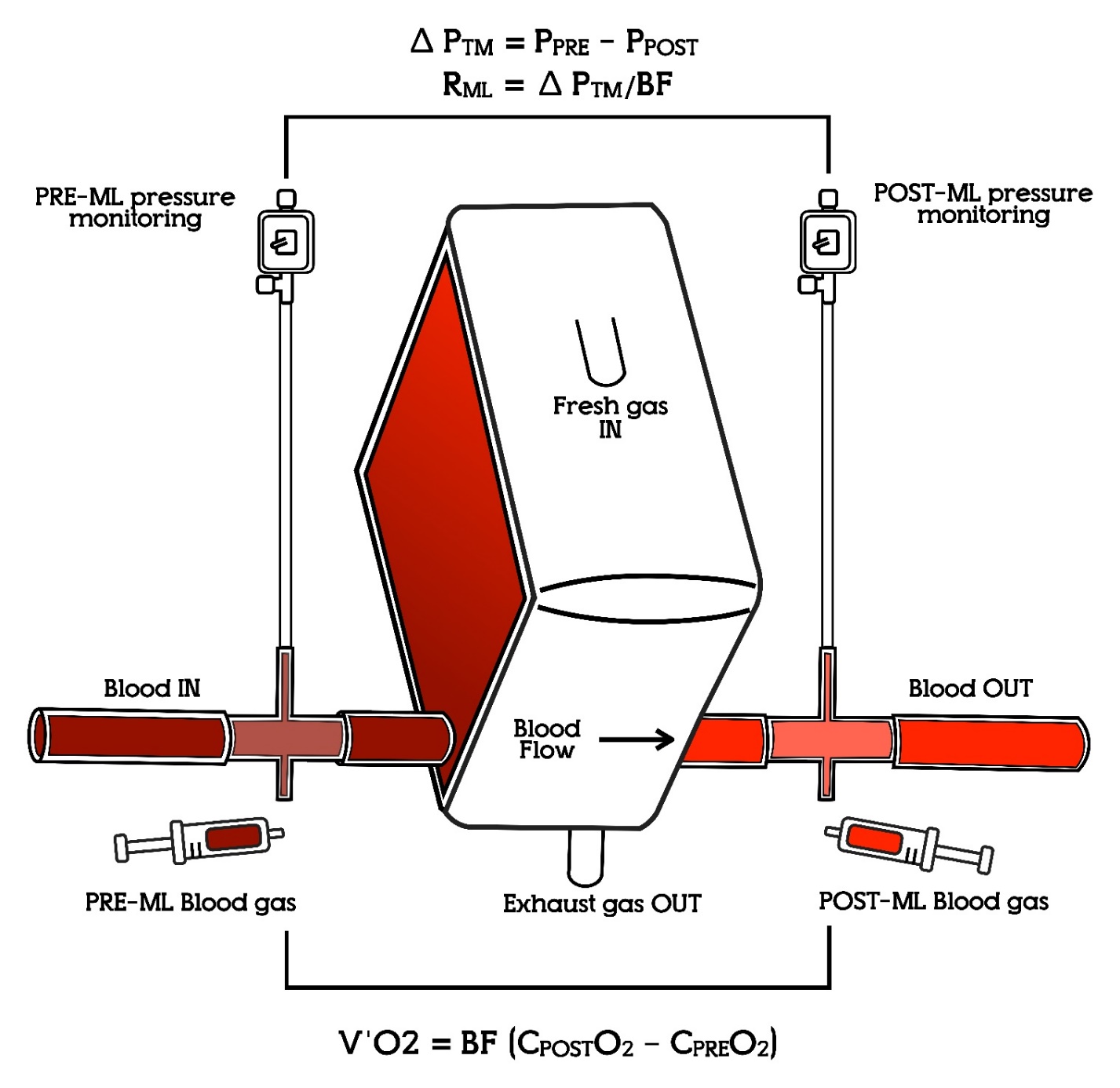


**Supplemental Figure.** Membrane lung monitoring of pressure drop and oxygen transfer. The pressure drop (ΔP) is calculated as the difference between the pre- and the post-ML pressure. The resistance within the ML, R_ML_, can be trended as ΔP / circuit blood flow rate (BFR). Oxygen transfer (V’O_2_) across the ML can be obtained from blood gas analysis as the product of BFR and the difference in oxygen content between the post- and pre-ML (C_Post_O_2_ – C_Pre_O_2_). Some ECMO consoles have integrated sensors that display pressure monitoring and blood oxygen saturations levels.
