# Supplementary material for: How I approach membrane lung dysfunction in patients receiving ECMO
Source: Crit Care. 2020 Nov 30;24:671. doi: 10.1186/s13054-020-03388-2 (PMC7704102; doi:10.1186/s13054-020-03388-2)
Supplement: Supplementary file 2 — Additional file 2. Sudden Membrane Lung Failure. [file 13054_2020_3388_MOESM2_ESM.docx]

**Sudden Membrane Lung Failure**

While serial monitoring of the ML may identify early markers of dysfunction and allow for elective exchange, acute unanticipated ML failure is a potentially life-threatening event with unique considerations. In these circumstances, rapid replacement of the obstructed ML may be necessary. However, logistical or organizational factors – such as lack of a pre-primed ECMO circuit or unavailability of an ECMO specialist primer – may delay such action, placing the patient at risk of rapid decline and death. A list of actions focused on preventing such events while also ensuring adequate preparation is provided in the Supplemental Table.

| **Sudden Membrane Lung Failure** | |
| --- | --- |
|  |  |
| **Prevention** | For persistent drainage insufficiency, consider echocardiography or CT scan (if feasible) to rule out thrombosis of drainage cannula or drainage vessel |
|  | In pro-thrombotic and pro-inflammatory states, consider increased intensity anticoagulation targets and frequent assessment of hematologic (coagulation and hemolysis) and inflammatory (cytokine) parameters |
|  |  |
| **Preparation** | Ensure immediately available and proximal pre-primed ML or circuit |
|  | Define emergency ventilator settings and hemodynamic support measures (including cardiac massage in VA ECMO-dependent patients) to be applied when emergently coming off ECMO support and while waiting for expeditious ML exchange |
|  | Perform multi-disciplinary hands-on training of emergency ML and circuit exchange |

**Supplemental Table**. Considerations to prevent and to prepare for sudden membrane lung failure. CT = computed tomography, ECMO = extracorporeal membrane oxygenation
